# Does oral cannabidiol oil in adjunct to pain medications help reduce pain and improve locomotion in dogs with osteoarthritis?

**DOI:** 10.18849/ve.v10i1.701

**Published:** 2025-03-27

**Authors:** Tracy Yeung, Eduardo Uquillas

**Keywords:** analgesia, cannabidiol, cannabinoid, cannabis, canine chronic pain, degenerative joint disease, osteoarthritis, pain management, pain relief

## Abstract

**PICO question:**

In dogs with osteoarthritis (OA), does the oral supplementation of cannabidiol (CBD) oil, compared to conventional treatment alone, improve treatment outcomes of reducing pain and improving locomotion?

**Clinical bottom line:**

**Category of research:**

Treatment.

**Number and type of study designs reviewed:**

Four papers were critically reviewed. Two of the studies were prospective, randomised, placebo-controlled, double-blind, cross-over clinical trials. One trial was a prospective clinical trial. One study was a prospective, randomised, controlled, clinical trial.

**Strength of evidence:**

Weak.

**Outcomes reported:**

The analgesic effect of CBD oil supplementation on dogs with OA, as assessed by different parameters. These parameters included pain scoring systems (Canine Brief Pain Inventory (CBPI): comprised of the Pain Severity Score and Pain Interference Score (PIS), Liverpool Osteoarthritis in Dogs (LOAD), and veterinarian assessment), activity assessments (Hudson activity scale, Activities of Daily Living (ADLs): based on Cincinnati Orthopaedic Disability Index (CODI), informal gait analysis, and objective gait analysis), and Quality of Life Index (QoL).

**Conclusion:**

CBD oil oral supplementation displayed a significant effect of extra pain relief on top of conventional treatment of canine OA in the clinical trials based on subjective pain assessments. However, the only study that evaluated pain and activity using objective measurements did not show significant improvements between treatment groups; therefore, the evidence supporting its use as an adjuvant to conventional therapy remains weak. Further studies utilising objective measurements are needed to improve the strength of the supporting evidence for a general use of CBD oil as additional analgesia for dogs with OA.

## How to apply this evidence in practice

The application of evidence into practice should take into account multiple factors, not limited to: individual clinical expertise, patient’s circumstances and owners’ values, country, location or clinic where you work, the individual case in front of you, the availability of therapies and resources.

Knowledge Summaries are a resource to help reinforce or inform decision-making. They do not override the responsibility or judgement of the practitioner to do what is best for the animal in their care.

## The evidence

Four clinical trials were reviewed to evaluate the analgesic effect of CBD oil oral supplementation alongside conventional medications for OA pain management (Brioschi et al., 2020; Gamble et al., 2018; Kogan et al., 2020; Mejia et al., 2021). Three out of the four reviewed studies showed a significant effect of CBD oil in reducing pain and improving activity of dogs based on subjective pain and activity scoring systems by owners and veterinarians (Brioschi et al., 2020; Gamble et al., 2018; Kogan et al., 2020). The remaining study is the first of the appraised studies to adopt objective gait analysis and it displayed no significant improvement of locomotion in the canine patients comparing those who received CBD oil treatment and the placebo group (Mejia et al., 2021). Currently, the evidence supporting the efficacy of use of CBD oil for relieving canine OA pain on top of conventional treatments of anti-inflammatories and other analgesics is weak.

## Summary of the evidence

**Brioschi et al. (2020):** Oral Transmucosal Cannabidiol Oil Formulation as Part of a Multimodal Analgesic Regimen: Effects on Pain Relief and Quality of Life Improvement in Dogs Affected by Spontaneous Osteoarthritis **Aim: **To assess the efficacy in pain management over a 12-week period of oral transmucosal cannabidiol (CBD), in combination with a multimodal analgesia, in dogs affected by spontaneous osteoarthritis (OA).

Population	Client-owned dogs presented to the Veterinary Teaching Hospital of the University of Milan (Lodi, Italy), for evaluation and treatment of pain related to osteoarthritis (OA) without underlying diseases and not receiving physical therapy. Dogs that did not receive anti-inflammatory medications and/or other analgesic therapies or undergo orthopaedic procedures within 4 weeks prior to the initial evaluation. 12 different breeds of dogs are included in the study. Weights ranged from 10–60 kg. Ages ranged from 7–2 years old. Affected joints included unilateral, bilateral or multiple shoulder, elbow, hip, and/or stifle. Initial patient screening and evaluation: physical examination, complete blood count (CBC) and serum biochemical analysis. Radiographic findings and OA localisation were recorded by a radiologist.
Sample Size	A total of 21 dogs completed the study. Three dogs were withdrawn from the study due to diagnosis of diseases and owners’ incompliance. The timing of each dog’s removal (at the time of recruitment or during the study) was not specified.
Intervention Details	Conventional treatment: All dogs received an anti-inflammatory drug—either firocoxib (non-steroidal anti-inflammatory drugs (NSAIDs)) or prednisone (glucocorticoids (GCs)), and analgesic drugs, including both gabapentin and amitriptyline. Firocoxib: Week 1: standard dose 5 mg/kg per os (PO) once daily (SID). Week 2: 2.5 mg/kg PO SID. From week 3 to week 12: 1.25 mg/kg PO SID. Prednisone: Week 1: standard dose 0.5 mg/kg PO twice daily (BID). Week 2: 0.25 mg/kg PO BID. From week 3 to week 12: 0.12 mg/kg PO BID. Gabapentin: Week 1: standard dose 10 mg/kg PO BID. From week 2 to week 12: 5 mg/kg PO BID. Amitriptyline: 1 mg/kg PO SID throughout the 12-week study period. Random allocation into treatment groups: CBD oil group (n = 9). Control group (n = 12)—without treatment at all, including placebo. Dogs in each group received the corresponding treatments for 12 weeks. Form of intervention: 2 mg/kg of CBD oil BID via oral transmucosal (OTM) route. Galenic formulation of CBD oil prepared and sold in authorised pharmacies. The CBD oil preparation was medium chain triglycerides oil containing 40, 100, or 200 mg/mL of CBD according to the patient weight. Other cannabinoids were of trace amounts (< 0.01 mg/mL). Dosing regimen: OTM CBD (2 mg/kg) BID was given by inserting a syringe without a needle into the buccal pouch. Performed by owner.
Study design	Prospective randomised controlled clinical trial.
Outcome studied	Pain measurement: Pain assessment recorded by owner based on: Canine Brief Pain Inventory (CBPI) scoring system—Pain Severity Score (PSS) and Pain Interference Score (PIS) (rating 0–10). Overall assessment of the dog’s quality of life—Quality of Life Index (QoL) (discrete numerical scale of 0 to 4). Recorded before treatment initiation and then at 1, 2, 4, and 12 weeks thereafter. Other measurements: CBC. Serum biochemistry analysis. Observations for gastrointestinal signs, ptyalism, somnolence, ataxia were recorded at treatment initiation and at the end of study.
Main findings (relevant to PICO question)	Considering similar baseline PSS, PIS, and QoL between the groups, significant reduction (P < 0.05) in PSS and PIS, and a significant increase (P < 0.05) in QoL was achieved in dogs who received OTM CBD oil (2 mg/kg BID) in addition to a multimodal analgesic regimen, compared to the findings of control group. Mean PSS (0–10) at weeks 0, 1, 2, 4, and 12: CBD group: 5.33 (SD= 2.4), 2.66 (SD = 1.6), 3 (SD = 1.2), 3.22 (SD = 1.5), 3.66 (SD = 1.4). Control group: 5.83 (SD = 2.2), 6.58 (SD = 1.8), 5.3 (SD = 2.0), 5.33 (SD = 2.0), 4.92 (SD = 2.1). Mean PIS (out of 10) at weeks 0, 1, 2, 4 and 12: CBD group: 6.33 (SD = 2.2), 2.44 (SD = 1.4), 3 (SD = 1.1), 4.33 (SD = 1.6), 2.44 (SD = 1.1). Control group: 7.25 (SD = 1.9), 6.66 (SD = 1.7), 6.41 (SD = 2.2), 5.25 (SD = 2.1), 6.33 (SD = 2.3). Mean QoL (out of 4) at weeks 0, 1, 2, 4 and 12: CBD group: 2.55 (SD = 0.7), 3.55 (SD = 0.5), 3.11 (SD = 0.6), 3.22 (SD = 0.6), 3.44 (SD = 0.7). Control group: 2.25 (SD = 0.8), 2.08 (SD = 0.9), 2.58 (SD = 0.9), 2.66 (SD = 1.0), 2.83 (SD = 0.9). In the CBD group, the decrease of PSS and PIS and the increase of QoL were significant over time. OTM CBD was well-tolerated by the dogs in the study in general and mild or absent gastrointestinal side effects occurred. 2/9 dogs in CBD group displayed minimal ptyalism. 1/9 dog in CBD group and 2/12 dogs in control group showed somnolence and mild ataxia. No relevant changes noted in the CBC and serum biochemical analysis in either the CBD or control groups at the end of the 12-week study.
Limitations	No justification for sample size selection, such as a power analysis. No placebo was given to the control group, meaning owners were aware of the treatment their dogs were receiving, leading to a potential placebo effect in the treatment group. Pain assessments were purely based on owners’ judgement, which could be subjective and biased. There was no medical follow-up in between the 12-week study period. The dose of anti-inflammatory drugs was adjusted based on owners’ judgement of pain level of their dogs. The subjectivity might lead to inappropriate reduction which negatively impacted the welfare and wellbeing of the dog; or inadequate reduction if the owner overestimated the pain level of the dog. The dogs in the study presented OA lesions in different joints such as shoulders, elbows, hips, stifles and with different degrees of injury showing different levels of radiographic evidence. The distribution of receptors CBD acts on, for example, transient receptor potential (TRP) cation channels that are related to pain regulation, and CB_1_, CB_2_, and opioid receptors, where CBD acts as allosteric modulator and indirect antagonist, are uneven in the body, so the effect of CBD might vary at different locations. Although blood count and biochemistry were measured, there were not evaluated statistically, considering previous papers have shown oral CBD oil administration might be associated with the increase of alkaline phosphatase (ALP) it would be useful to obtain data on the correlation of OTM CBD oil use and ALP level in the study alongside were unknown. This added a confounding factor to the outcome. The study is short-term (12 weeks) and the long-term effect of CBD was not investigated.

Table note...

**Gamble et al. (2018):** Pharmacokinetics, Safety, and Clinical Efficacy of Cannabidiol Treatment in Osteoarthritic Dogs **Aim: **To determine oral pharmacokinetics and assess safety and analgesic efficacy of a cannabidiol (CBD) oil in dogs with osteoarthritis (OA).

Population	Client-owned dogs presented to Cornell University Hospital (USA) for evaluation and treatment of a lameness due to osteoarthritis (OA) without other underlying diseases and not receiving physical therapy. This paper includes two different studies including a pharmacokinetic study and a clinical trial. Two different populations were involved in these two studies. Pharmacokinetic study: Male beagles 3.5–7 years, male castrated, 10.7–9 kg. Clinical trial: 8 different breeds of dogs are included in the study. Weights ranged from 17.6–50 kg. Ages ranged from 3–14 years old. Affected joints included unilateral, bilateral or multiple spine, shoulder, elbow, carpus, hip, and/or stifle. Initial patient screening and evaluation: complete blood count (CBC), serum biochemical analysis, radiographic examination of affected joints, owner questionnaire defining the affected limb(s), duration of lameness, and duration of analgesic or other medications taken. The affected limb(s), duration of lameness, and duration of analgesic or other medications taken were recorded.
Sample Size	Pharmacokinetic study: 4 male beagles. Clinical trial: 16 dogs completed the trial. 6 dogs were removed from the study due to diagnosis of diseases and. The timing of each dog’s removal (at the time of recruitment or during the study) was not specified.
Intervention Details	Pharmacokinetic study: Each of the four dogs in the study received a 2 mg/kg and an 8 mg/kg oral dosage of CBD oil, with a 2-week washout period in between. 5 ml of blood was collected at time 0, 0.5, 1, 2, 4, 8, 12, and 24 hours after oil administration. Clinical trial: Conventional treatment: All dogs were fed their regular diet with no change allowed during the trial. Dogs were allowed only to receive non-steroidal anti-inflammatory drugs (NSAIDs), fish oil, and/or glucosamine/chondroitin sulfate without any changes to the doses of these medications for 4 weeks prior to or during the 10-week study. Concurrent NSAIDs treatment used in the dogs in the study: 6 dogs were on carprofen (2 mg/kg once daily (SID)—2.4 mg/kg twice daily (BID)) 3 dogs were on meloxicam (0.1 mg/kg SID) 7 dogs did not receive NSAID treatment. Other analgesic medications used, such as gabapentin and tramadol, were discontinued at least 2 weeks prior to enrolment. Random allocation into treatment groups: Cannabidiol (CBD) oil group (n = 9). Placebo oil group (n = 7). Patients received either CBD or placebo treatment for a period of 4 weeks. A two-week washout period was allowed between treatments. Followed by another 4 weeks of crossing over to the opposite treatment. Form of intervention: Volume of CBD oil each dog received was calculated according to the weight of each dog (2 mg/kg of CBD oil BID). Final desiccated product from proprietary hemp strain produced by ethanol and heat extraction that was reconstituted into an olive oil base. About 10 mg/ml of CBD oil as an equal mix of CBD oil and cannabidiolic acid (CBDa). Other cannabinoid (CB) components in the CB preparation: 24 mg/ml tetrahydrocannabinol (THC) 27 mg/ml cannabichromene (CBCe) 11 mg/ml cannabigerol (CBG) All other cannabinoids were less than 0.01 mg/ml. Less than a 9% difference in concentration of CB components across batches in five productions. Form of placebo: Equivalent volume (calculated according to the weight of each dog) of olive oil with 10 parts per thousands of anise oil and 5 parts per thousands of peppermint oil giving a similar herbal smell.
Study design	Two-part study, a pharmacokinetic study and a clinical trial. The clinical trial is a prospective, randomised, placebo-controlled, owner and veterinarian double-blind, cross-over clinical trial.
Outcome studied	Pharmacokinetic study: Maximum concentration (ng/ml) of CBD oil in the blood. Time of maximum concentration of CBD oil (hour) reached in the blood. Half-life of elimination of CBD oil (hour). Area under the curve (time 0–24 hours) (ng–hr/ml). Median residence time (hour) of CBD oil in the blood. Clinical trial: Pain measurement: Veterinarian: an ordinal scoring system according on lameness, pain on palpation, and weight bearing. Owner used the canine brief pain inventory (CBPI) (0–40) and the Hudson activity scale (0–110) before treatment initiation and at weeks 2 and 4 thereafter. Other measurements: Serum biochemistry analysis was used at treatment initiation and at weeks 2 and 4 thereafter.
Main findings (relevant to PICO question)	Pharmacokinetic study: Median maximal concentration of serum CBD was reached after 1.5 hours for 2 mg/kg CBD dose at 102.3 ng/ml (60.7–0 ng/ml; 180 nM) and after 2 hours for 8 mg/kg CBD dose at 590.8 ng/ml (389.5–904.5 ng/ml; 1.2 uM). The terminal half-life of this oil-based oral hemp preparation in which CBD was the most abundant cannabinoid (2 mg/kg per os (PO) BID), was between 4 and 5 hours. Clinical trial: Dogs with OA receiving this industrial hemp extract high in CBD (2 mg/kg of CBD BID) were perceived to have decreased pain and increased activity (P < 0.01) at weeks 2 and 4 post-CBD treatment comparing to week 0. Week 0 median CBPI pain: 21/40 (± 8); CBPI activity interference 35/60 (± 15); Hudson 54/110 (± 13). Week 2 median CBPI pain: 14/40 (± 6); CBPI activity interference 25/60 (± 15); Hudson 67/110 (± 15). Week 4 median CBPI pain: 14/40 (± 8); CBPI activity interference 26/60 (± 14); Hudson 67/110 (± 10). No statistical significances were observed across treatment. A decrease in veterinary pain scores in the CBD group was shown when compared to baseline on evaluation at week 2 (P < 0.01) and week 4 (P < 0.02). No significant changes in CBC values in both CBD and placebo groups over the period of the study. 9/16 dogs showed significant increase in alkaline phosphatase (ALP) over time from baseline by week 4 in the CBD oil treatment group (P < 0.01). Week 0 mean ALP 160 (± 212) U/L. Week 2 mean ALP 238 (± 268) U/L. Week 4 mean ALP 323 (± 407) U/L. Alanine aminotransferase (ALT) and aspartate transferase (AST) were normal in all dogs throughout the study. No other significant changes in serum biochemistry values in both CBD and placebo groups during study. No observable side effects or clinical signs in the dogs underwent OA treatment during the study.
Limitations	Initial power analysis was performed (power 0.80; alpha of 0.05): 14 dogs were required to detect significant differences in outcomes of interest. Although the requirement was met by having 16 dogs in the clinical trial, the sample size was considerably small. The study is short-term (2 treatments of 4 weeks each), and the long-term effect of CBD was not investigated. The dogs in the study presented OA lesions in different joints and with different degrees of injury showing different clinical signs, CBPI and Hudson scores. The distribution of receptors CBD acts on, for example, transient receptor potential (TRP) cation channels that are related to pain regulation, and CB_1_, CB_2_, and opioid receptors, where CBD acts as allosteric modulator and indirect antagonist, are uneven in the body, so the effect of CBD might vary at different locations. The dogs participated in the study were receiving different conventional treatments, such as different doses and types of NSAIDs, in addition to potential fish oil and glucosamine/chondroitin sulfate, during the treatment. The drug interactions between the cannabinoids and the medications or supplements given alongside were unknown. This added a confounding factor to the outcome.

Table note...

**Kogan et al. (2020):** The Use of Cannabidiol-Rich Hemp Oil Extract to Treat Canine Osteoarthritis-Related Pain: A Pilot Study **Aim: **To assess the impact of a full-spectrum cannabidiol (CBD) oil on dogs with chronic pain as a result of osteoarthritis (OA).

Population	Patients were recruited from a specialised animal pain management clinic in Colorado, USA. The recruitment of the remaining 5 dogs was not mentioned. Dogs with chronic osteoarthritis (OA) pain for at least 3 months in duration. Dogs with owners who were interested in trying a cannabidiol (CBD) product for pain management of their dogs. Dogs with owners who were able to commit to the 90-day study with dogs’ medical assessments every 2 weeks. Dogs with owners who were willing to keep an informal journal of their dogs’ activities of daily living (ADLs) using the Cincinnati Orthopaedic Disability Index (CODI) as a guide during the duration of the study to better understand the impact of the CBD product. Dogs with owners who agreed not to use any medications or supplements that were not approved by the veterinarian performing the assessments during the 90-day course of the study. 19 different breeds of dogs are included in the study. Weights ranged from 5–50 kg. Ages ranged from 2–6 years old. Initial patient screening and evaluation: full physical exam (pain palpation and mapping of pain patterns), informal gait analysis (observation of location and severity of lameness under different movements assessed by veterinarian), complete blood count (CBC), serum biochemical analysis and screening thyroid profile.
Sample Size	A total of 32 dogs completed the trial. Only 5 dogs did not complete the study because of medical conditions or owners’ withdrawal. The timing of each dog’s removal (at the time of recruitment or during the study) was not specified.
Intervention Details	Conventional Treatment: Specific pain-directed medications that were permitted during study included gabapentin and polysulfated glycosaminoglycan (PSGAG) without any changes to the dosing regimen during the study. For the dogs taking gabapentin for chronic maladaptive pain at the time of study enrolment, once their comfort level was stable following CBD dose escalations, gabapentin dose reductions were attempted. Depending on the patients’ conditions, the dosing of gabapentin ranged from 10–40 mg/kg q 8–12 hours in order to provide adequate analgesia. When attempted to reduce gabapentin dose, 20–40% of the total daily dose was reduced at each adjustment and the dosing frequency was adjusted. Other pain therapies such as medical acupuncture, therapeutic laser, and nutraceuticals were evaluated on a case-by-case basis The use of non-steriodal anti-inflammatory drugs (NSAIDs), tramadol or amantadine were restricted from all participant. Form of intervention: Certified organic, cold-pressed hemp seed oil infused with 1000 mg of full-spectrum hemp extract derived from organically grown hemp plants cultivated in Colorado. Full-spectrum extract includes cannabinoids (such as cannabidiolic acid (CBDa), CBD, cannabigerol (CBG), cannabichromene (CBCe)), flavonoids, terpenes, and other constituents within the cannabis plant. 87 mg/ml of CBD. Other cannabinoid components in the cannabinoid preparation: 23 mg/ml tetrahydrocannabinol (THC) 13 mg/ml tetrahydrocannabinolic acid A (THC-A) 84 mg/ml CBG The other cannabinoids were less than level of detection (LOD). Dosing regimen and adjustments: CBD oil preparation at a dose of 0.25 mg/kg delivered on food once daily (SID) for 3 days and then twice daily (BID) for the rest of the study. CBD dose escalations of 0.5 to 0.75 mg/kg approximately every 12 hours were prescribed at each reassessment until the patient’s pain score on palpation was 0 to 1 on a scale of 10.
Study design	Prospective clinical trial.
Outcome studied	Pain measurement: Pain scale of 0–10 was given by a single veterinarian based on: Physical exam: pain palpation and mapping of pain patterns. Informal gait analysis: observation of location and severity of lameness under different movements assessed by veterinarian. Owners’ records on dogs’ ADLs using the CODI. Before treatment initiation followed by biweekly assessments during the study for dose adjustments. Other measurements: Serum biochemistry (alanine aminotransferase (ALT), alkaline phosphatase (ALP)) recorded at the study’s initiation and end.
Main findings (relevant to PICO question)	30 out of the 32 dogs that completed the trial had significantly improved pain scores (P < 0.001) or had a consistent pain score at 0/10 to 2/10 with a reduced dose of gabapentin after receiving CBD oil supplementation. Changes in numeric pain score (0–10) pre- & post-treatment: −2.23 ± 2.3 (P < 0.001). Changes in gabapentin dose (mg/day) pre- & post-treatment: −1263 ± 1314 (P < 0.001). Both non-responders were King Charles Spaniels. The dose of CBD needed to achieve a positive analgesic effect ranged from 0.3–4.12 mg/kg BID. Dogs that underwent gabapentin dose reduction had a reduced dose of 20–60% of the original dose. The level of ALP, but not ALT, increased significantly during the 90-day CBD treatment. Initial mean ALP: 133.3 (± 118) U/L. Final mean ALP: 264 (± 233.2) U/L. Mean increase of ALP 130.8 (± 135) U/L.
Limitations	No justification for sample size selection was given, such as a power analysis. There were no control groups and owners were aware of the treatment their dogs were receiving, leading to a potential placebo effect. The participation of the study is voluntary and dog owners with interests in trying CBD products are more likely to sign up. In addition to the study not being blinded and used owners’ subjective assessment for pain level of dogs, there is a higher likelihood of the placebo effect affecting the pain assessment. The location of OA in the dogs which might affect CBD efficacy were not documented. The distribution of receptors CBD acts on, for example, transient receptor potential (TRP) cation channels that are related to pain regulation and CB_1_, CB_2_, and opioid receptors, where CBD acts as allosteric modulator and indirect antagonist, are uneven in the body, so the effect of CBD might vary at different locations. Only the dose and strategy for dose reduction of gabapentin as a conventional treatment were presented but not the PSGAG. As there could be potential drug interactions and affect the study outcome, dose, and dosing regimen of conventional treatment alongside CBD supplementation should be presented as reference. The dogs participated in the study were receiving different conventional pain-specific medications, such as different dose of gabapentin and PSGAG during the study. The drug interactions between the cannabinoids and the medications or supplements given alongside were unknown. This added a confounding factor to the outcome. CBD oil doses were increased until the pain score of subject decreases and gabapentin doses were reduced when analgesia was considered adequate in subjects which was subjectively assessed. The doses of the CBD oil and conventional pain relief were not fixed in individual subjects throughout the study, leading to unreliability of final pain score as marker of treatment success with subjects receiving different doses of treatment. The study is short-term (90 days), and the long-term effect of CBD was not investigated.

Table note...

**Mejia et al. (2021):** Evaluation of the Effect of Cannabidiol on Naturally Occurring Osteoarthritis-Associated Pain: A Pilot Study in Dogs **Aim: **To assess the safety and effect of cannabidiol (CBD) for symptom relief of canine osteoarthritis (OA)-associated pain in a clinical setting using objective outcome measures.

Population	Dogs that were not on concurrent treatment with any cannabis product at the time of evaluation. Dogs that did not undergo any previous orthopaedic surgical procedure or any intra-articular injection within 6 months before enrolment. Dogs that did not exhibit palpable instability of the shoulder or stifle joint (dogs with chronic, stable cranial cruciate ligament disease were eligible). Dogs with Canine Brief Pain Inventory (CBPI)—pain severity score (PSS), and pain interference score (PIS) of at least 2 out of 5. Dogs with radiographically confirmed OA within 6 months of enrolment and was consistent with the observed lameness. Dogs presented a subjectively identifiable lameness (at least 2 and < 5 on a 0–5 scale evaluated by a veterinarian). Client-owned dogs with naturally occurring osteoarthritis (OA) presented to the Colorado State University Veterinary Teaching Hospital, USA without underlying diseases. 12 different breeds of dogs are included in the study. Ages ranged from 4–14 years old. Weights ranged from 22–63 kg. Affected joints included unilateral, bilateral or multiple elbows, hip, and/or stifle. Initial patient screening and evaluation: physical examination, complete blood count (CBC) and serum biochemical analysis.
Sample Size	A total of 23 dogs completed the trial. Four dogs were removed from the study due to health complications, adverse effects after CBD administration, and owner incompliance. The timing of each dog’s removal (at the time of recruitment or during the study) was not specified.
Intervention Details	Conventional treatment: Dogs were allowed only to receive NSAIDs treatment throughout the study period if they were under consistent NSAID therapy previously. The same administration regimen remained throughout the study. Concurrent NSAIDs treatment used in the dogs in the study: 9 dogs were on carprofen. 2 dogs were on meloxicam. 1 dog was on grapiprant. 11 dogs did not receive NSAID treatment. The use of new medications, supplements, dose changes or other treatment strategies during the study were disallowed. Random allocation into treatment groups: CBD-placebo group (n = 11). Placebo-CBD group (n = 12). 4 weeks of baseline measurements. 6 weeks of either CBD or placebo treatment depending on the subject’s group. Followed by another 6 weeks of crossing over to the opposite treatment. Form of intervention: 5 mg/kg twice daily (BID) of CBD oil. Plant-derived CBD oil made from highly purified CBD product adding into cold-pressed hemp seed oil and chicken flavouring. Composed predominantly of CBD but also included small amounts of other cannabinoids, including tetrahydrocannabinol (THC), cannabidiolic acid (CBDa), cannabinol (CBN) and cannabigerol (CBG) (specific concentrations not reported). < 0.3% THC by dry weight. Form of placebo: Cold-pressed hemp seed oil and chicken flavouring without the addition of the highly purified CBD product.
Study design	Prospective, randomised, placebo-controlled, double-blind, cross-over clinical trial.
Outcome studied	Pain measurement: Owner: Liverpool Osteoarthritis in Dogs (LOAD) and CBPI. Between weeks 1 and 4 during the baseline period, at week 10 (cross-over point), and at week 16 (end of the study). Objective measurements: Accelerometry—total activity counts (ACs) were measured using at least 1 of 2 different devices (Actical® or Heyrex®). The percentage of change in total AC was measured by the means from weeks 1–4 (baseline period) and weeks 1–3 and 4–6 for each of the two treatment periods for comparison between groups and over treatment time. Objective gait analysis—a pressure-sensitive walkway (PSW) was used, and the peak vertical force normalised by body weight (PVF%) and the percentage of body weight distribution (%BWD) was calculated. Objective gait analysis was performed once weekly during the baseline period (weeks 1–4), followed by every 3 weeks after initiation of the first treatment period. Other measurements: Serum biochemistry analysis: Initial and 6 weeks after CBD administration. Plasma CBD levels: At week 10 (cross-over point) and at the end of the study by a validated liquid chromatography-mass spectrometry-based assay.
Main findings (relevant to PICO question)	There were no significant differences (P > 0.05) noted between treatment groups at any time point for any of the recorded outcome measures in all the parameters including pain scoring by owners: CBPI PSS (P = 0.89). CBPI PIS (P = 0.59). LOAD (P = 0.74). Objective gait analysis – %BWD (p-value between treatment groups at week 3 = 0.24; p value between treatment groups at week 6 = 0.73). A significant improvement (P < 0.05) was detected over within the CBD treatment group for one of the ground reaction force measurements - %BWD (P within CBD group at week 3 = 0.0013; P within CBD group at week 6 = 0.05), but not PVF% (P within CBD group at week 3 = 0.085; P within CBD group at week 6 = 0.15). 14 out the 23 dogs who received CBD treatment experienced elevations of alkaline phosphatase (ALP), alanine aminotransferase (ALT) and /or aspartate aminotransferase (AST). This was the main adverse effect of CBD supplementation found in this study. The median plasma CBD levels after 6 week of CBD oil administration at 311 ng/ml (range 5–860) was higher than that after 6 weeks of placebo at 0.96 ng/mL (range 0.6–572) A wide range of plasma CBD levels in dogs was displayed in both groups after 6 weeks: CBD group 5–860 ng/ml. Placebo group 0.6–572 ng/ml.
Limitations	Post-hoc power analysis was performed (power 0.80; alpha of 0.05): 59 dogs were required to detect significant differences of 2.5% and 17 dogs were needed to detect significant differences of 5% in outcomes of interest. The sample size of this study was 23, so it was able to detect 5% significant differences. As no significant differences between treatment groups were detected in this study, future studies with a larger sample size allows a more precise detection of significant differences of 2.5%. The study is short-term (2 treatments of 6 weeks each) and the long-term effect of CBD was not investigated. The dogs in the study presented OA lesions in different joints and with different degrees of injury showing different clinical signs, LOAD, and CBPI scores. The distribution of receptors CBD acts on, for example, TRP cation channels that are related to pain regulation, and CB_1_, CB_2_, and opioid receptors, where CBD acts as allosteric modulator and indirect antagonist, are uneven in the body, so the effect of CBD might vary at different locations. The dogs who participated in the study were receiving different conventional treatments i.e. different doses and types of NSAIDs during the treatment. The drug interactions between the cannabinoids and the medications or supplements given alongside were unknown. This added a confounding factor to the outcome. Pain assessments were purely based on owners’ judgement which were subjective and could be biased. Hemp oil was used as the base for the placebo group so it might still have an effect on the subjects with its cannabinoid components in it despite low concentrations. Accelerometry data was not reported in the tables with the other objective gait analysis parameters.

Table note...

### Appraisal, application and reflection

Osteoarthritis (OA) is a common condition seen in dogs, prevalent in 20% of dogs over 1 year old in both the UK (Clements et al., 2006) and in North America (Johnston, 1997). Without a definitive cure, the treatment goals of OA focus on decelerating the disease progression (Martello et al., 2022) and the palliation of associated pain (Johnston et al., 2008), which improves patients’ daily activities. Current pain management strategies primarily involve the use of anti-inflammatory drugs, including non-steroidal anti-inflammatory drugs (NSAIDs) and glucocorticoids (GCs), in combination with analgesics such as gabapentin and amitriptyline (Brioschi et al., 2020). With research demonstrating the insufficiency of pain relief (Lascelles et al., 2008) and potential side effects (Brioschi et al., 2020; Gamble et al., 2018) of the NSAIDs and the lack of knowledge surrounding gabapentin and amitriptyline efficacy (Johnston et al., 2008), the discussion on canine OA-related pain management and the search for new treatments and therapies is still ongoing.

The minimally psychoactive cannabidiol (CBD), a class of cannabinoids (CBs) that acts on the endocannabinoid system (ECS), has demonstrated a great potential in providing extra analgesic effects (Landa et al., 2016; O'Brien & McDougall, 2018). Because CBD is lipophilic, and oral administration is the easiest and non-invasive route of administration, studies giving oral supplementation of CBD oil are the most abundant of the investigations into the efficacy of CBD use in dogs and there is, therefore, more evidence available for analysis.

Four papers were reviewed, and all had relatively small sample sizes and were short-term prospective studies. While all studies were able to assess the analgesic effects provided by the oral CBD supplementation by identifying changes in pain and activity scores in patients, only two papers (Gamble et al., 2018; Mejia et al., 2021) reported a power analysis. Statistical significance of the improvement in scores remains in question in Kogan et al. (2020) and Brioschi et al. (2020). More research is needed to give stronger statistical evidence to display correlations between the intervention and the improvement. Overall, a consensus was not reached by reviewing these four studies. Gamble et al. (2018), Kogan et al. (2020), and Brioschi et al. (2020) showed a positive effect of CBD oil in reducing pain and improving activity of dogs based on subjective pain and activity scoring systems by owners and veterinarians. Nevertheless, Mejia et al. (2021) was the first of the appraised studies to adopt objective gait analysis and it displayed no significant improvement of locomotion in the canine patients comparing those who received CBD oil treatment and the placebo group. Hence, the evidence supporting the pain-relieving effects of oral supplementation of CBD oil in dogs with OA remains weak.

Different CBD preparations were used in each study and there were variations in the concentration of the other CB components reported. In Gamble et al. (2018), the concentration of cannabidiolic acid (CBDa) was at a significant level of 1:1 to CBD with tetrahydrocannabinol (THC), cannabichromene (CBCe) and cannabigerol (CBG) at detectable levels in the preparation; with CBD as the main component, THC, tetrahydrocannabinolic acid A (THC-A) and CBG were at detectable levels in Kogan et al. (2020); CBD was the only significant component in Brioschi et al. (2020); concentration of CB products were not reported in Mejia et al. (2021). Constituents in the cannabis plant have synergistic and entourage effects (Anand et al., 2021) and a different combinations or formulations of the CBs might impact on the efficacy of the CBD preparation providing analgesia to the dogs. Although the three studies reported variable CB components concentrations all displayed an outcome of improved pain and activity in the patients after CBD administration (Brioschi et al., 2020; Gamble et al., 2018; Kogan et al., 2020), the entourage effects of CBs should be noted and taken into consideration for study design of future research.

The dogs in all studies presented OA lesions in different joints (unilateral, bilateral or multiple spine, shoulder, elbow, carpus, hip, and/or stifle) and with different degrees of injury showing different clinical signs and pain scores. The distribution of receptors CBD acts on, for example, transient receptor potential (TRP) cation channels that are related to pain regulation (Zou & Kumar, 2018), cannabinoid receptors CB_1_ and CB_2_ , where CBD acts as allosteric modulator and indirect antagonist, potentiating the effect of THC (Laprairie et al., 2015), and opioid receptors where CBD exhibits positive allosteric modulation (Kathmann et al., 2006), are uneven throughout the body, so the efficacy of CBD in relieving pain might vary at different locations. Potentially, painful areas with fewer CB receptors might need a higher dose of CBD for local analgesia. Different CB components work on different CB receptors, therefore different formulations of CB products might be used to treat pain at different locations. Further research more specific on the effect of CBD formulations on the CB receptors at targeted areas is required. Besides the location of OA, better study design is also needed for future research regarding variation of sizes, genetics, lifestyle/activity level, and diet of participating dogs. These factors should be controlled in future studies to minimise confounders affecting the effect of CBD supplementation.

The method of administration may also affect the absorption of CBD oil. Whilst three studies (Gamble et al., 2018; Kogan et al., 2020; Mejia et al., 2021) supplemented the subjects orally or into the food, CBD oil was inserted into the buccal pouch using a syringe in Brioschi et al., 2020. The absorption of the CBD via the oral transmucosal (OTM) route may be different to the oral route, which relies solely on alimentary absorption, with the addition of transmucosal absorption. This factor may affect the pharmacokinetics and hence the efficacy of the CBD oil.

While the papers showed variable effects of CBD oil supplementation for pain relief in canine patients with OA, one side effect reported in all four studies was the elevation of alkaline phosphatase (ALP) in serum biochemistry analysis after CBD administration. It is currently unknown whether it is a side effect of the CBD administration itself, or an effect of drug interactions with other medications as there is no study on CBD interactions with other drugs in a canine model yet (Gamble et al., 2018). Naturally extracted phytocannabinoids are known to inhibit and temporarily deactivate the liver cytochrome P450 system at a dose-dependent fashion (Copas et al., 2021). CBD administration may delay the metabolism and prolong activity of analgesic agents when it is given as an add-on to conventional analgesics and anti-inflammatory drugs. It potentiates the pain-relieving effect of conventional drugs but at the same time contributes to potential side effects by raising and prolonging a high plasma concentration of the conventional drugs which many of the drugs rely heavily on liver metabolism for elimination (Copas et al., 2021). Elevations of alanine aminotransferase (ALT) and aspartate aminotransferase (AST) were reported in Mejia et al. (2021). In spite of the absence of significant elevations of ALT and AST in Gamble et al. (2018) and Kogan et al. (2020), such elevation is a potential side of effect of CBD treatment, which could impact a patient’s liver. Investigations on CBD interactions with other medications and the safety of long-term or high-dose administration of CBD would help determine safe use of CBD products in multimodal pain management plan for canine patients suffering from OA pain.

Although the research question we try to answer aims to investigate the analgesic effect of CBD oil supplementation alongside conventional treatment compared to conventional pain relief alone, the conventional treatment is in fact a confounding variable. Drug interactions between CBD and medications commonly used for palliative pain relief for canine OA are not fully understood. Dogs in Gamble et al. (2018), Kogan et al. (2020), and Mejia et al. (2021) received different doses and types of anti-inflammatories and/or analgesics depending on the patient’s own condition during the study. Studies that offer the same conventional treatment, such as Brioschi et al. (2020), would eliminate this confounding factor; however, another problem arises with this approach—the baseline level of pain relief provided by the conventional drugs is different in each patient because of individual variations in the response to drugs due to pharmacogenetics. This produces another confounding factor when evaluating the effect of CBD supplementation in adjunct to conventional pain treatment.

Furthermore, monoclonal antibody therapy targeting at nerve growth factor, such as bedinvetmab in dogs, has gained much attention and popularity for OA pain management. As the treatment is relatively new in veterinary medicine, there is limited study on the analgesic effect of monoclonal antibodies alongside CBD oil. The knowledge gap of the interactions between the two therapies is likely to be closed in the future given their great potential in widespread use in veterinary analgesia.

Pharmacogenetics also contributed to variable responses to CBD administration. It was described in Kogan et al. (2020) that there were 2 ‘non-responders’ amongst the 32 dogs who did not show any changes to overall mobility and comfort during the study, with their overall pain scores remaining at 1/10 despite the fact that the CBD dose was increased along the course of the 90-day study. It is uncertain if it coincidental or a breed-related response as both ‘non-responders’ were King Charles Spaniels. This breed of dog was not included in the other three reviewed papers (Brioschi et al., 2020 ; Gamble et al., 2018; Mejia et al., 2021), disallowing further inquiry into the response of CBD in the breed.

Aside from the consideration of individual variations, pharmacokinetics are also relevant in the discussion of the efficacy of CBD oil as an oral supplementation for relieving OA pain. Plasma CBD concentrations were measured in Mejia et al. (2021) and a wide of range of plasma CBD levels in dogs was displayed (5–860 ng/ml) after 6 weeks of CBD administration at 2.5 mg/kg BID. A pharmacokinetic profile of CBD oil was produced by Gamble et al. (2018), nevertheless, the entourage effect of different CB components in different formulations may influence the pharmacokinetics of the CBD oil in individual animals. Better pharmacokinetic understanding is required for the justification of general use of CBD products for canine OA pain treatment.

Multiple modal pain management involves the interplay of treatments to act on one or more pain pathways for pain alleviation for the patient. It is a challenging task to assess the effectiveness of a single component of a multimodal pain management plan. Despite the weak evidence supporting the pain-relieving effects of oral supplementation of CBD oil in dogs with OA, there are still many gaps in our knowledge for its therapeutic use. Success in individual animals shows it has its potential to provide pain relief and it is an option for some patients to trial. In fact, CB products currently available commercially in Australia are mostly hemp seed oil without specifying concentrations of its CB constituents. The prices range widely from $16 to $180 per 100 ml. The unclear labelling of effective CB components and highly variable prices further contribute to push factors for dog owners when considering trials of CBD to aid managing their dogs’ OA pain. Afterall, the general use of CBD oil oral supplementation for dog as pain relief for OA remains doubtful due to variable responses in patients. Pain management strategies could be different for individuals. CBD use has a great potential in individualised medicine before the acquisition of knowledge to use it as a general analgesic for OA pain. Trials and testing allow fine adjustments of dose and administration which help in creating a long-term pain management plan to best-fit owners’ and patients’ lifestyles and permit better control and management of potential side effects of CBD by close monitoring.

In conclusion, CBD oil oral supplementation resulted in extra pain relief, in addition to the relief given by conventional treatment of canine OA in the clinical trials that were based on subjective pain assessments only (Brioschi et al., 2020; Gamble et al., 2018; Kogan et al., 2020). The only study that evaluated pain and activity using objective measurements reviewed in this Knowledge Summary did not show significant improvements between treatment groups (Meija, 2021). Because of this, the evidence supporting its use as an adjuvant to conventional therapy is still weak. More studies that utilise objective pain measurements are needed in the future. The evaluation of the use of CBD products in the multimodal pain management plan for dogs with OA would also be aided by further investigations of drug interactions between CBD and conventional OA pain medications and relevant potential side effects of CBD administration, pharmacogenetics and variation of responses to CBD in different patients, pharmacokinetic profiles of different CB formulations considering the entourage effect of different CB components, and the potential of alternate administration routes of CBD instead of oral in order to produce a more stable CBD concentration level in patients and hence better improve pain and activity levels. Although there is little evidence to back up the general use of CBD oil oral supplementation alongside conventional treatment to provide additional analgesics, CBD oil is an option with its potential for individualised pain management. Trials and fine adjustments of dose and administration route may enable optimal combinations of pain management strategies matching with owners’ and patients’ lifestyles and better control over potential side effects of CBD administration, leading to better animal well-being, welfare, and quality of life.

### Methodology

**Search Strategy:** 

Databases searched and dates covered	CAB Abstracts via Web of Science 1910 to June 2024 Medline via OVID 1946 to June 2024 PubMed via NCBI interface 1900 to June 2024
Search terms	CAB Abstracts: ((TS=(dog*) OR TS=(canine)) AND (TS=(osteoarthritis) OR TS=(degenerative arthritis) OR TS=(degenerative joint disease*)) AND (TS=(cannabidiol*) OR TS=(cannabis*) OR TS=(cannabinoid*) OR TS=(hemp*))) AND (TS=(convention*) OR TS=(treat*) OR TS=(therap*) OR TS=(anti-inflammator*) OR TS=(non-steroidal anti-inflammator*) OR TS=(NSAID*) OR TS=(glucocorticoid*) OR TS=(corticosteroid*) OR TS=(glucocorticosteroid*) OR TS=(analgesi*) OR TS=(gabapentin*) OR TS=(amitriptyline*)) AND (TS=(pain*) OR TS=(manag*) OR TS=(relief*) OR TS=(reliev*) OR TS=(analgesi*)) Medline: ((Dogs/ or dog*.mp.) or (canine.mp.)) and ((osteoarthritis.mp. or Osteoarthritis/) or (degenerative arthritis.mp. or Osteoarthritis/) or (degenerative joint disease*.mp.)) and ((Cannabidiol/ or cannabidiol*.mp.) or (Cannabis/ or cannabis*.mp.) or (cannabinoid*.mp.) or (hemp*.mp.)) and ((convention*.mp.) or (treat*.mp.) or (therap*.mp.) or (anti-inflammator*.mp.) or (Anti-Inflammatory Agents/ or Anti-Inflammatory Agents, Non-Steroidal/ or non-steroidal anti-inflammatory.mp.) or (NSAID*.mp.) or (glucocorticoid*.mp.) or (Glucocorticoids/ or corticosteroid*.mp.) or (analgesi*.mp. or Analgesics/) or (Gabapentin/ or gabapentin*.mp.) or (Amitriptyline/ or amitriptyline*.mp.)) and ((pain*.mp. or Pain/) or (Pain Management/ or pain management.mp.) or (analgesi*.mp. or Analgesics/)) PubMed: ((dog*) OR (canine)) AND ((osteoarthritis) OR (degenerative arthritis) OR (degenerative joint disease*)) AND ((cannabidiol*) OR (cannabis*) OR (cannabinoid*) OR (hemp*)) AND ((convention*) OR (treat*) OR (therap*) OR (anti-inflammator*) OR (non-steroidal anti-inflammator*) OR (NSAID*) OR (glucocorticoid*) OR (corticosteroid*) OR (glucocorticosteroid*) OR (analgesi*) OR (gabapentin*) OR (amitriptyline*)) AND ((pain*) OR (manag*) OR (relief*) OR (reliev*) OR (analgesi*))
Dates searches performed:	03 June 2024

Table note placeholder

**Exclusion / Inclusion Criteria:** 

	Exclusion / Inclusion criteria
Exclusion	Papers that are not peer-reviewed. Papers not written in the English language. Papers that did not answer the PICO question. Papers that did not have their study subjects on conventional pain medications at the time of CBD oil supplementation. Papers not published in the last six years.
Inclusion	Papers having their study subjects as dogs with OA condition.

Table note placeholder

**Search Outcome:** 

Database	Number of results	Excluded – not published in the last 6 years	Excluded – not primary research	Excluded – did not answer the PICO question	Excluded – subjects did not receive conventional pain medications	Total relevant papers
CAB Abstracts	29	3	11	10	1	4
MEDLINE	18	2	8	3	2	3
PubMed	22	2	11	4	2	3
Total relevant papers when duplicates removed						4

Table note placeholder

### ORCiD

Tracy Yeung: https://orcid.org/0009-0005-7664-0770

Eduardo Uquillas: https://orcid.org/0000-0002-4227-2173

### Conflict of Interest

The author declares no conflicts of interest.
